# Malignant pleural mesothelioma with long-term tumor disappearance of a local relapse after surgery: a case report

**DOI:** 10.1186/1752-1947-3-6800

**Published:** 2009-03-27

**Authors:** Masahiko Higashiyama, Kazuyuki Oda, Jiro Okami, Jun Maeda, Ken Kodama, Fumio Imamura

**Affiliations:** 1 Department of Thoracic Surgery, Osaka Medical Center for Cancer and Cardiovascular Diseases, 1-3-3 Nakamichi, Higashinariku, Osaka, 537-8511, Japan; 2 Department of Respiratory Medicine, Osaka Medical Center for Cancer and Cardiovascular Diseases, Osaka, Japan

## Abstract

**Introduction:**

There have been few reports of spontaneous regression of malignant pleural mesothelioma, but the mechanism for this is unknown. We present a case report on a patient with malignant pleural mesothelioma showing apparent tumor disappearance in a local relapse after surgery.

**Case presentation:**

A 73-year-old man presented with malignant pleural mesothelioma in the right thoracic cavity. A pleurectomy was performed, and as expected, the tumor locally relapsed with increasing chest pain. However, the symptoms suddenly improved while the tumor was apparently reduced, and spontaneous tumor regression was initially considered. The patient confessed that he had self-administered a mushroom extract with alternative parasympathetic nerve stimulation therapy thereafter. The complete disappearance of the tumor was clinically achieved during a 29-month follow-up with continuing self-treatment.

**Conclusion:**

This is the first report describing a malignant pleural mesothelioma patient in Japan showing long-term complete disappearance of a local relapse after surgery. This event was a tumor regression possibly due to an immunological effect of combined complementary and alternative therapy.

## Introduction

Although the standard therapy for malignant pleural mesothelioma (MPM) is still undetermined, the major therapeutic modality for this disease is surgery, radiation and chemotherapy. The majority of cases are at an advanced stage, thus several novel modalities to improve the overall survival time have been preliminarily explored. Immunotherapy, molecular-targeted therapy, and gene therapy are candidate therapies, but cases of long-term survival are exceptional.

In spite of the advanced-stage disease, complete or marked regression of MPM has been described [1]–[4]. These surprising events are mostly due to chemotherapy achieving complete remission [1], and only three reports have described spontaneous regression of this disease [2]–[4]. Recently, a patient with MPM experienced a complete tumor regression of a local relapse after cytoreduction surgery. It is possible that this unique favorable event was due to the effect of combined complementary and alternative self-therapy.

## Case presentation

**Figure 1:**
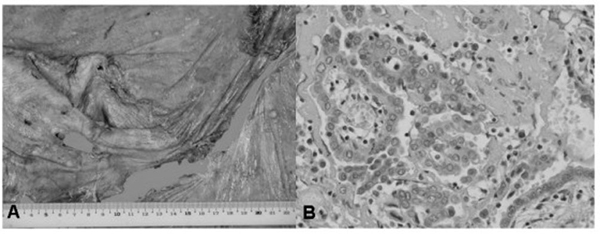
**Macroscopic (A) and microscopic (B) findings of the surgically resected malignant pleural mesothelioma**.

A 73-year-old man with a 75-pack-year history of cigarette smoking and asbestos exposure between the ages of 30 and 40 years had been admitted to undergo an extrapleural pneumonectomy due to MPM in the right pleural cavity. However, only a cytoreduction pleurectomy was performed on 30 September 2003 (Figure 1A), because of the aggressiveness of the local tumor. The lesion remained mainly in the mediastino-hilar region adjacent to the carina, esophagus, and the right main bronchus. Histologically, the tumor was epithelioid type (Figure 1B) with T4N0, stage IV (IMIG staging). Then, postoperative intrathoracic chemothermotherapy using carboplatin (CBDCA, 450mg intrapleurally, one course) was administered, followed by systemic chemotherapy using gemcitabine (GEM, 0.8mg/m^2^, biweekly, 6 courses). Chest computed tomography (CT) in December 2003 showed that the effect of these postoperative therapies on the residual tumor was stable disease (SD).

**Figure 2:**
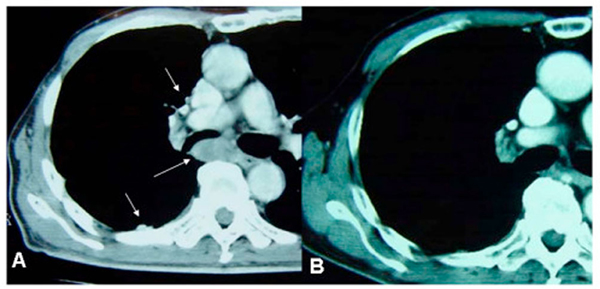
**Local relapse before treatment (A) and tumor disappearance after treatment (B) on chest computed tomography**.

In May 2004, the patient felt increasing chest pain with poor general condition. Chest CT showed local relapse broadly in the right pleural cavity causing airway narrowing (Figure 2A). However, he refused further chemo-radiation therapy, and in June 2004, without consulting with the physicians, he orally self-administered a mushroom extract containing Agaricus blazei Murill Kyowa (ABMK) [5], in addition to alternative parasympathetic nerve stimulation therapy in another hospital. This is a modified acupuncture modality providing possible immune-modulation [6]. After experiencing high fever for about 2 weeks, his general condition distinctly improved. Four months after these therapies, the relapsed bulky tumor in the pleural cavity had significantly decreased, and finally completely disappeared on chest CT (Figure 2B). Then, the patient continued this self-treatment with neither symptoms nor radiological evidence of tumor relapse in May 2007. Tumor disappearance was completely achieved during a 29-month follow-up.

Unfortunately, local relapse was detected on chest CT in August 2007. In November 2008, although the relapsed tumor was again growing slowly, the patient was alive while continuing this self-treatment.

## Discussion

The median survival times for patients with an unresectable or postsurgical recurrent MPM are usually reported to be in the 6- to 12-month range with the best supportive care, and even now, most chemotherapeutic regimens have shown no or only a minor benefit to the survival rate. In this patient, although an extrapleural pneumonectomy was initially selected as the first step, only a cytoreduction pleurectomy was performed. Therefore, postoperative treatment including intrathoracic chemothermotherapy and systemic chemotherapy was positively administered yielding SD, but unfortunately, a local re-growth of the tumor occurred later. Surprisingly, considering the usually poor prognosis of this disease, the present clinical course after a local relapse seems unique. It is extremely interesting to elucidate the mechanism of regression of the tumor.

Initially, the disappearance of the tumor was viewed as a result of the delayed effect of postoperative chemotherapy; however, by reviewing the clinical course and condition of the patient, this judgment was found to be negative. In addition, since the patient had taken non-steroidal anti-inflammatory drugs (NSAIDs) continuously after undergoing a pleurectomy, it also seemed that this medication had had little effect on the observed tumor regression. Next, a so-called "spontaneous regression" of the tumor was considered, because the patient did not reveal that he had received the "complementary or alternative combined therapy". Spontaneous regression of MPM has been described in only three reports [2]–[4]. A clinical summary of these reported cases is shown in Table 1. According to these reports, spontaneous regression of MPM may be strongly associated with lymphocyte-mediated immunity. Robinson *et al.*[2] emphasized an association between MPM regression and some immunological mechanism based on the histological observation of massive lymphoid infiltration within the tumor tissue. Pilling *et al.*[3] also reported similar histological findings. In our patient, however, such histological evidence was not seen in the surgically resected tissue.

**Table 1:** Reported cases of spontaneous regression of malignant pleural mesothelioma

Reporter Country	Year	Gender	Age	Histology	Previous therapy	Patient Time to regression	Outcome	Mechanism
						Period of regression		
Robinson et al. [2] Australia*	2001	Female	54	Mixed	No	3 months	Died	Immunological reaction?
				(with lymphoid infiltration)		Unknown		
Pilling et al. [3] UK**	2007	Male	58	Epithelioid	Surgery	Unknown	Survival without relapse	Host response
				(with inflammatory response)		7 years		
Allen RKA [4] Australia**	2007	Female	61	Epithelioid	No	6 months	Survival without relapse	Immunological reaction?
				(poorly differentiated)		5 years		
The present case Japan**	2009	Male	73	Epithelioid	Surgery	4 months	Survival with re-relapse	
					Chemotherapy	29 months	Complementary and alternative therapy?	

*Case of spontaneous marked regression

**Cases of spontaneous complete regression

Thirdly, after having revealed this "hidden combined therapy", tumor disappearance could be rather considered as a "therapeutic effect" of achieving complete remission. ABMK, a mushroom extract, is considered a health food in many countries after it was reported to be a potential source of anti-tumor, anti-metastatic, cytotoxic and immunoactive compounds [5,7]. Experimentally, Kimura *et al.*[7] showed that some substances isolated from ABMK inhibited tumor growth through the mechanism of both anti-angiogenic and immune-modulatory activity. Ahn *et al.*[5] reported that natural killer cell activity was clinically elevated by ABMK-treated gynecological cancer patients. Another therapy, parasympathetic nerve stimulation therapy with a minor modification using a laser machine, is widely performed as alternative therapy for patients suffering from cancer as well as various other types of disease in Japan [6]. In particular, for cancer-bearing patients, it was said that acupuncture therapy could provide a beneficial effect in anti-cancer treatment by enhancing the cellular immune function [8]; however, so far, there has been no report describing the clinically complete remission of malignancy by these therapies.

Alternative, but more scientific, immunotherapy has been clinically explored to treat MPM [9]. One is specific immunotherapy which targets particular antigens in MPM tissue, and the other is a non-specific, but anti-tumor immunotherapy using such cytokines as interleukin 2 (IL-2), tumor necrosis factor (TNF), and interferon (INF) [9]. In fact, complete remission of MPM was experienced by INF administration through intra-pleural administration [10].

In our patient, considering that the timing of the improvements in his general condition after a high fever and tumor disappearance accorded with the influence of this "complementary or alternative treatment", it is likely that this successful clinical outcome resulted in complete remission. However, it is unknown whether the AGMK or parasympathetic nerve stimulation or both combined brought about the most favorable effect, and importantly, there are no scientific grounds to confirm the direct effect of this treatment. Several immunological blood parameters such as serum IL-2, INF-alpha, INF-gamma, and CD4/CD8 ratio were examined after the tumor disappearance, but all were within the normal range (data not shown).

In summary, this report presents a patient with MPM with a clinical tumor disappearance after a local relapse during a 29-month follow-up period. The mechanism of this tumor disappearance could not be sufficiently explained. Importantly, the mechanism of spontaneous regression of this disease in previous reports [2]–[4] is considered to be strongly associated with some immunological reaction, and the good effect of such complementary or alternative treatment modalities [5]–[8] is also caused by a similar immune response. Considering these data together, some immunological reactions of the host to the tumor are thus suggested to be responsible in this patient.

## Conclusion

This is the first report describing a MPM patient in Japan showing long-term complete disappearance of a local relapse after surgery. The mechanism of this surprising tumor disappearance cannot be categorically explained. However, the clinical course suggests that some immunological reactions of the host to the tumor may be responsible.

## Abbreviations

ABMK: Agaricus blazei Murill Kyowa; CBDCA: carboplatin; CT: computed tomography; GEM: gemcitabine; IL-2: interleukin-2; IMIG: International Mesothelioma Interest Group; INF-alpha: interferon-alpha; INF-gamma: interferon-gamma; MPM: malignant pleural mesothelioma; SD: stable disease; TNF: tumor necrosis factor.

## Consent

Written consent was obtained from the patient for publication of the case report and any accompanying images. A copy of the written consent is available for review by the Editor-in-Chief of the journal.

## Competing interests

The authors declare that they have no competing interests.

## Authors' contributions

MH conceived the study concept and design, was involved with patient care and drafted the manuscript and literature review. KO, JO, JM: conceived the study concept and design, were involved with patient care and drafting the manuscript. KK: was involved with formation of the study concept and design and drafting the manuscript, FI: was involved with formation of the study concept and design, patient care and drafting of the manuscript and literature review. All authors have read and approved the final version of the manuscript.

## References

[B1] Umsawasdi T Dhingra HM Charnsangavej C Luna MA A case report of malignant pleural mesothelioma with long-term disease control after chemotherapy Cancer 1991 67 48 54 10.1002/1097-0142(19910101)67:1<48::AID-CNCR2820670110>3.0.CO;2-I 1898706

[B2] Robinson BW Robinson C Lake RA Localised spontaneous regression in mesothelioma - possible immunological mechanism Lung Cancer 2001 32 197 201 10.1016/S0169-5002(00)00217-8 11325491

[B3] Pilling JE Nicholson AG Harmer C Goldstraw P Prolonged survival due to spontaneous regression and surgical excision of malignant mesothelioma Ann Thorac Surg 2007 83 314 315 10.1016/j.athoracsur.2006.05.070 17184695

[B4] Allen RKA Apparent spontaneous complete regression of a multifocal malignant mesothelioma of the pleura MJA 2007 187 413 415 17908008 10.5694/j.1326-5377.2007.tb01315.x

[B5] Ahn WS Kim DJ Chae GT Lee JM Bae SM Sin JI Kim YW Namkoong SE Lee IP Natural killer cell activity and quality of life were improved by consumption of a mushroom extract, Agaricus blazei Murill Kyowa, in gynecological cancer patients undergoing chemotherapy Int J Gynecol Cancer 2004 14 589 594 10.1111/j.1048-891X.2004.14403.x 15304151

[B6] Mori H Nishijo K Kawamura H Abo T Unique immunomodulation by electro-acupuncture in humans possibly via stimulation of the autonomic nervous system Neurosci Lett 2002 320 21 24 10.1016/S0304-3940(02)00012-5 11849754

[B7] Kimura Y Kido T Takaku T Sumiyoshi M Baba K Isolation of an anti-angiogenic substance from Agaricus blazei Murill: Its antitumor and antimetastatic actions Cancer Sci 2004 95 758 764 10.1111/j.1349-7006.2004.tb03258.x 15471563 PMC11159378

[B8] Wu B Effect of acupuncture on the regulation of cell-mediated immunity in the patients with malignant tumors Zhen Ci Yan Jiu 1995 20 67 71 8758833

[B9] Schwarzenberger P Byrne P Kolls JK Immunotherapy-based treatment strategies for malignant mesothelioma Curr Opin Mol Ther 1999 1 104 111 11249674

[B10] Boutin C Nussbaum E Monnet I Bignon J Vanderschueren R Guerin JC Menard O Mignot P Dabouis G Douillard JY Intrapleural treatment with recombinant gamma-interferon in early stage malignant pleural mesothelioma Cancer 1994 74 2460 2467 10.1002/1097-0142(19941101)74:9<2460::AID-CNCR2820740912>3.0.CO;2-N 7923001

